# Evaluation of Microlenses, Color Filters, and Polarizing Filters in CIS for Space Applications

**DOI:** 10.3390/s23135884

**Published:** 2023-06-25

**Authors:** Clémentine Durnez, Cédric Virmontois, Pierre Panuel, Aubin Antonsanti, Vincent Goiffon, Magali Estribeau, Olivier Saint-Pé, Valérian Lalucaa, Erick Berdin, Franck Larnaudie, Jean-Marc Belloir, Catalin Codreanu, Ludovic Chavanne

**Affiliations:** 1 Centre National D’Etudes Spatiales (CNES), 18 Avenue Edouard Belin, 31400 Toulouse, France; 2 Department of Electronics, Optronics and Signal Processing (DEOS), Institut Supérieur de l’Aéronautique et de l’Espace (ISAE-SUPAERO), 10 Avenue Edouard Belin, 31400 Toulouse, France; 3 Airbus Defence and Space, 31 Rue des Cosmonautes, 31400 Toulouse, France

**Keywords:** CMOS image sensors, space application, microlenses, polarizing filter, color filter array, optical stack, transmittance, quantum efficiency

## Abstract

For the last two decades, the CNES optoelectronics detection department and partners have evaluated space environment effects on a large panel of CMOS image sensors (CIS) from a wide range of commercial foundries and device providers. Many environmental tests have been realized in order to provide insights into detection chain degradation in modern CIS for space applications. CIS technology has drastically improved in the last decade, reaching very high performances in terms of quantum efficiency (QE) and spectral selectivity. These improvements are obtained thanks to the introduction of various components in the pixel optical stack, such as microlenses, color filters, and polarizing filters. However, since these parts have been developed only for commercial applications suitable for on-ground environment, it is crucial to evaluate if these technologies can handle space environments for future space imaging missions. There are few results on that robustness in the literature. The objective of this article is to give an overview of CNES and partner experiments from numerous works, showing that the performance gain from the optical stack is greater than the degradation induced by the space environment. Consequently, optical stacks can be used for space missions because they are not the main contributor to the degradation in the detection chain.

## 1. Introduction

CMOS image sensors have risen to have very high performance during the last two decades, thanks to the commercial market. Indeed, CMOS technology was first pushed to the mobile phone market, where the goal has been to shrink the pixel to reach large pixel arrays. Then the machine vision, security and automotive markets pushed the technology even more in order to reach very high performances. All of these trends have forced engineers to create specific technology bricks. In order to improve their performances, optical components can be added to the pixel stack: microlenses [1], color filters [2], and/or polarizing filters [3]. These components are commonly used for commercial purposes, but their robustness to a harsh environment such as space has been hardly studied.

Space imaging missions require high-performance image sensors. Thanks to their good electro-optical performances, high integration level, low power consumption, and tolerance to the space radiation environment, CMOS image sensors (CIS) are more and more preferred over Charge Coupled Devices (CCD) in many current space imaging missions, such as for the rover MARS 2020 Perseverance [4,5], One Web constellation [6], and selected on MMX [7]. 

The detection chain of a space imaging system is one of the main elements and contributes to the performance of the system. In case of degradation, the imaging system can have a huge impact on image acquisition and thus the interpretation of the observed scene. This is why image sensors are exposed to very harsh constraints before being embedded on a space mission. The goal of this article is to evaluate if the additional components in the optical stack can affect the required detection chain performances. For this purpose, a wide range of devices from various commercial CMOS foundries and featuring different optical stacks are analyzed. This will also allow us to review, gather and compare a large amount of results in a single document.

The goal of this article is to show that the use of an additional optical stack is suitable for a large majority of space applications, regardless of the type, technology, and foundry.

First, experimental details will be given: the device’s characteristics as well as environmental tests will be detailed, and the optical stack properties will be introduced. The possible degradation mechanisms and how the tolerance of the devices is analysed will be explained.

Then, the results over the wide range of devices will be exposed. Not all tests are performed under the same conditions and with the same setup, which makes comparisons based on environmental tests difficult. Therfore the results are presented foundry by foundry and a conclusion can thus be drawn for each of them. A discussion is provided to show the global behavior of this technology independentely of the foundry.

Finally, a discussion on the relevance of the use of the optical stack for a given space mission is provided.

## 2. Experimental Details

In order to demonstrate the tolerance of CIS optical stacks to the space environment, samples have been exposed to a wide range of constraints. Experimental tests have been implemented to measure the induced degradation. The aim of this section is to detail the devices used in this study, as well as their main characteristics, and to present the experimental setup and measurements performed to investigate the potential degradation. 

### 2.1. Devices

Results from 15 references of CIS from 5 different foundries have been compiled. Their characteristics are reported in Table 1. The investigated technological nodes range from 65 to 180 nm, and the pixel pitch of the devices ranges from 1.1 to 52 µm, with both Front-Side Illuminated (FSI) and Back-Side Illuminated (BSI) sensors. Some of the devices are equipped with both microlenses and Color Filter Array (CFA) (A.1, C.1, C.2, D, E), some are equipped with microlenses only (A.2, B.1, B.3, D, F) or CFA only (B.5, B.6). Device C.3 presents the same pixel architecture as device C.1, but the CFA is replaced by an array of polarizing filters with different polarization angles. Devices without any specific optical stack have been used for comparison.

### 2.2. Optical Stacks

Optical stacks are widely used for ground purposes because they can provide a huge gain in detector performance for several markets (mobile phone, security machine vision, automotive…). Several types of optical stacks exist: organic microlenses can increase the collection of impinging photon, or filters can provide selectivity of photon or polarization of the light. This is why the different optical stacks used in this study are described below.

#### 2.2.1. Microlenses

**Figure 1:**
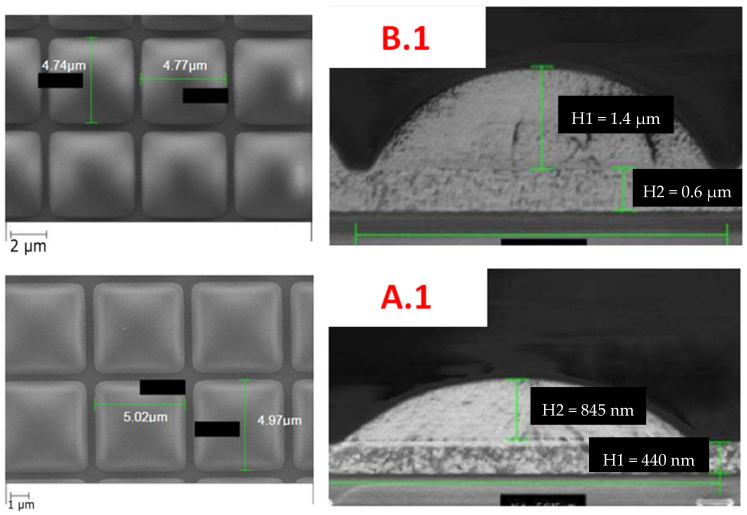
SEM top and side views of microlenses of device A.1 (multilayer polymer 595 nm) and B.1 (single-layer polymer 440 nm).

Microlenses are used to focus light into the photosensitive volume of pixels, thus increasing the efficiency of photon collection. In this study we focus on organic lenses. Scanning Electron Microscopy (SEM) top and side views of the microlenses of devices A.1 and B.1 can be seen in Figure 1. 

**Figure 2:**
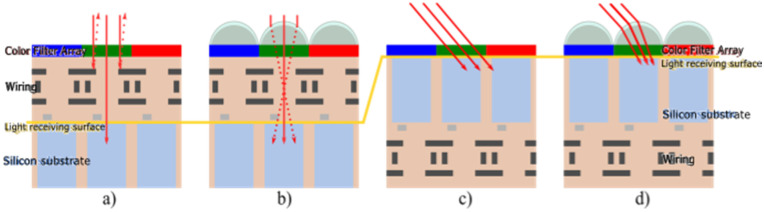
FSI CIS with (**b**) and without (**a**) microlenses, showing the focus of light into the photosensitive volume, and BSI CIS with (**d**) and without (**c**) microlenses, illustrating the reduction in optical crosstalk.

In Front-Side Illuminated (FSI) global shutter devices, focusing light away from the pixel’s collecting node also lowers the Parasitic Light Sensitivity (PLS), as shown in [8]. In Back Side Illuminated (BSI) global shutter CIS, microlenses also help decrease the PLS, but they also improve the Modulation Transfer Function (MTF) by reducing the optical crosstalk [9], as illustrated in Figure 2.

The material used to process these microlenses is organic, mainly transparent polymer, such as Polymethyl Methacrylate (PMMA) or similar. Modern consumer CIS often integrates several layers of microlenses in its optical stack [1].

Microlenses are widely used for consumer applications, but their robustness regarding harsh environments has to be demonstrated before embedding them in space missions. Possible degradation would be a clouding of the material, which would thus decrease the transmittance and inhibit the collection of incident light.

Some advanced technologies use a specific architecture named the light pipe. This system guides the light to the pixel photosensitive element using a top organic microlens and a dielectric microlens etched in the optical stack under the first one [10]. 

#### 2.2.2. Color Filter Array (CFA)

**Figure 3:**
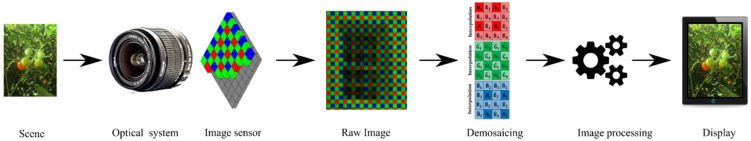
Illustration of the acquisition of a color image using a CIS with a Color Filter Array based on an RGB Bayer matrix.

A Color Filter Array (CFA) consists of tinted resins or a glass array placed on top of a sensor’s pixel array following a specific pattern. CFAs are commonly used in consumer applications (cameras, smartphones) in order to sample the spectral response of a scene in a single frame, unlocking a very user-friendly color imaging, as presented in Figure 3 with a simple Green–Blue–Red–Green Bayer pattern [11]. Thanks to this element, the user can obtain a colored image at a high frame rate. Moreover, last-generation demosaicing algorithms allow one to retrieve almost the full resolution of an image, with color [12].

In space applications, CFAs have been used inside high-resolution context cameras, such as the Remote Micro-Imager (RMI) in the SuperCam instrument aboard the Mars 2020 Perseverance rover, which upgraded ChemCam’s RMI onboard Curiosity by providing color capability [4]. 

Some new technologies are also based on resin, such as in [13] where chrominance luminance studies are presented.

Other optical systems, such as Fabry–Perot resonators, can be used to sample the spectral response of a scene, but they are not included in the scope of this study. Such sensors are called multispectral or hyperspectral because they allow sampling of the light spectrum with a fine spectral resolution [14,15]. 

**Figure 4:**
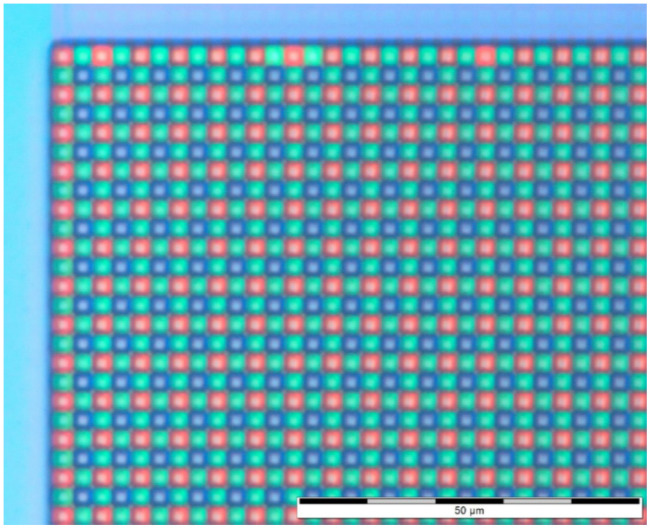
Microscope view of device D, showing the RGB Bayer matrix.

Figure 4 represents a microscope optical image of CIS D, which contains a Bayer CFA.

#### 2.2.3. Polarizing Filters

The polarized nature of incident light cannot be detected by a conventional image sensor. In typical consumer applications, sensing polarized light reveals scratches and stains on a homogenous glass plane, showing distortion due to mechanical constraints, differentiating materials, or removing light reflections [3]. 

**Figure 5:**
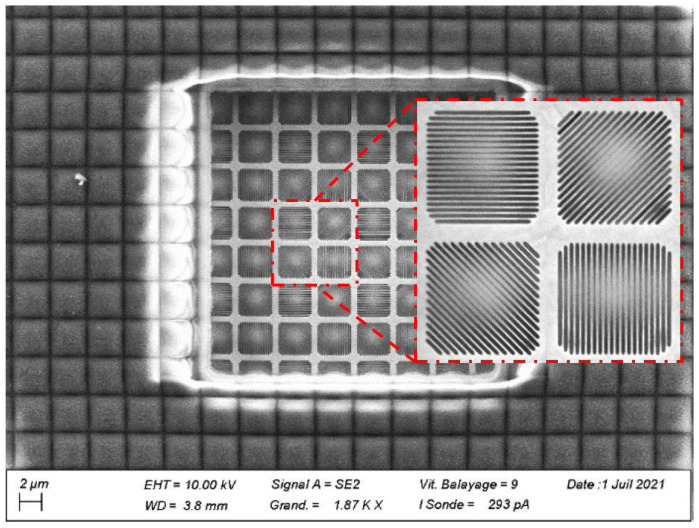
SEM view of image sensor C.3.

For space applications, it could help to decrease specular reflections, spot specific materials on the ground, or help to identify the approach of incoming foreign objects such as space debris. To be able to detect the polarization of the light, a sensor can be equipped with a four-directional polarizing grid formed on-chip under the microlenses layer, as seen in Figure 5, which represents a SEM top view of device C.4 after a Focused Ion Beam cut. A tungsten layer, etched in order to create the polarizing filter array, can be seen under the microlenses layer.

### 2.3. Measurements

In order to assess the performances of the optical parameters or the degradation due to the exposition of harsh constraints, an experimental setup has been implemented. Parameters that can illustrate the performance are detailed, as well as the expected degradation. 

#### 2.3.1. Studied Parameters 

CIS are complex devices, and several parameters can be measured to precisely characterize these sensors. The goal is to obtain the highest signal-to-noise ratio and a good uniformity to achieve the good performance of the instrument. The signal consists of quantum efficiency (QE), Charge to Voltage Factor (CVF), and gains. The noise consists of dark signal noise, flickers noises, and readout chain noise as per the EMVA 1288 standard.

The CVF is obtained thanks to the Photon Transfer Curve (PTC) [16]. The Electro Optical Transfer Function (EOTF) gives the photoresponse in function of incident light flux for a given wavelength. Quantum efficiency is defined as the ratio between collected charges number and incoming photons number, which corresponds to the slope of EOTF in the linear part with photoresponse converted in electrons thanks to CVF and gains [17].

(1) $$QE=\frac{number\;of\;collected\;electrons}{number\;of\;incident\;photons}=\frac{1}{CVF}\frac{v}{Nph}$$

Hence, a diminution of the efficiency of the pixel microlenses and/or filters will directly affect the QE. 

**Figure 6:**
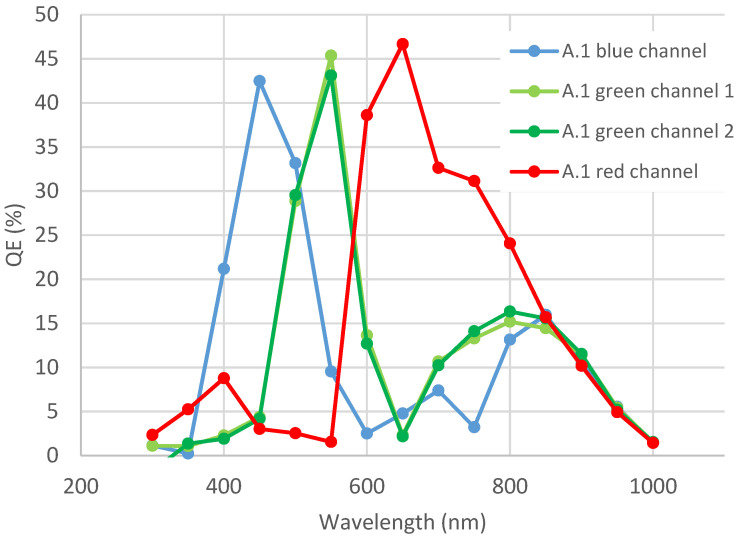
QE curve in function of wavelength for device A.1 containing an RGB filter. These measurements have been performed before any environmental test.

In this study, the focus is set on quantum efficiency parameter, plotted in function of the wavelength of incident light. This measurement is particularly interesting for samples with RGB filters. For example, Figure 6 shows the curve for sample A.1, with RGB filters.

**Figure 7:**
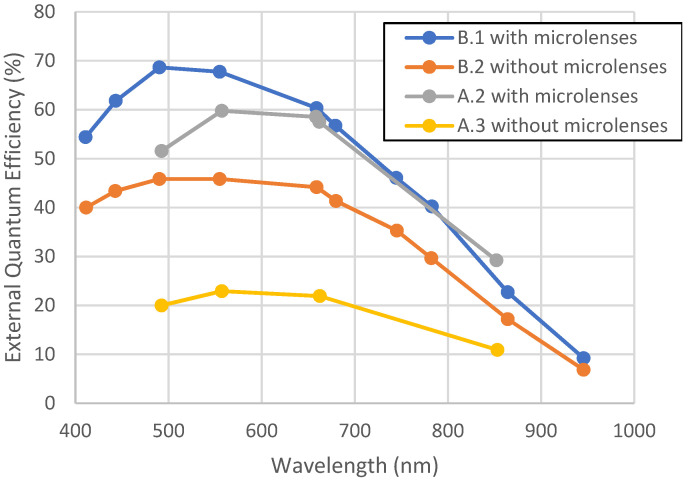
Quantum efficiency in function of wavelength for several devices from foundry A and B (Front-Side Illuminated), illustrating the improvement due to microlenses. These measurements have been performed before any environmental test.

Figure 7 represents pristine results of QE before any environmental test. It can be seen that the QE has been increasing by a factor of 55% for devices from foundry B and 140% for devices from foundry A thanks to the addition of microlenses. For these devices, the pixel pitch is under 7 µm, which means that the pixels are small, and thus they collect less signal. This shows that under this pitch of 7 µm, microlenses are required in order to keep a good QE.

When the same detector is available with or without an optical stack, the degradation can directly be measured through the transmittance, which is expressed as the ratio between photoresponse of pixels with optical stack and photoresponse of pixels without optical stack for the same incident flux.

#### 2.3.2. Environmental Tests

Satellite and instruments are operated in a very harsh environment. All the microelectronic technologies used for these missions have to be evaluated and qualified in order to confirm their tolerance to space environment.

First, all of them are exposed to screening as per ESCC 9020 in order to remove all infant mortalities (accelerate early failure) and bring the component to constant failure. Moreover, some samples (not flight devices but devices from the same batch) are exposed to harsh constraints in order to ensure that systems will operate until the end of the mission. Most of test methods detailing the procedure and expected impacts are described in standards, such as MIL-STD-883-1:

- Thermal cycling: this test consists of submitting samples alternatively to extreme high and low temperatures for a certain amount of time. This corresponds to strong thermal gradients for several cycles. This allows us to determine the evolution of optical properties of materials and the occurrence of delamination, cracks, outgassing, and interface weakening.
- Humidity or moisture test: samples are exposed to a given amount of humidity, reflecting worst-case conditions that devices could handle during ground storage. This reveals distortion, corrosion, material clocking, or swelling in case of humidity absorption.
- High-Temperature Storage (HTS): samples are exposed to a high temperature for a long time (usually several thousands of hours) in order to ensure long-term reliability and outgassing troubles. This corresponds to accelerated aging.
- Vacuum test: devices exposed to vacuum can suffer from layers ungluing or outgassing.
- Radiations: samples are exposed to several types of particles representative of space conditions. Indeed, devices cannot be protected by the atmosphere, and they are directly exposed to particles and high energetic photons from radiation belts and solar eruptions, leading to cumulative effects such as Total Ionizing Dose (TID) or Total Non-Ionizing Dose (TNID). In order to ensure tolerance to space radiations, devices are usually exposed to gamma (Cobalt 60) for ionizing effects and protons for both ionizing and non-ionizing effects. Sometimes, X-rays may also be used to evaluate robustness with regard to ionizing doses. TID can induce ionization of the material, and TNID can modify the crystalline structure, both leading to the modification of optical properties, such as darkening [18].
- UV test: this test evaluates the tolerance to UV from the sun due to the lack of Ozone layer. Polymers can thus be ionized, leading to darkening of materials [19].
- Cleaning: devices may be cleaned during system integration because of molecular or particle contamination. For this purpose, acetone and IPA ethanol are evaluated in order to determine if they can be used without any effects on the sensors.

Therefore, in order to ensure that optical stack is tolerant to space environment, several types of tests have been performed in the frame of several R&Ds and projects. Table 2 summarizes all the environmental tests that have been conducted on our large set of data.

In this study, the different tests can reveal the following physical degradation on the image sensor optical stack.

- Thermal cycling: Interfaces between optical stack and silicon can be weakened because of ungluing, leading to a worse charge collection.
- Humidity: humidity absorption can lead to the modification of layers or distortion of filters/microlenses. This would decrease the charge collection efficiency. Moreover, pollution and outgassing can induce unwanted particles in front of optical stack, which would also prevent charges from being collected.
- High-temperature storage: The evolution of optical properties can be followed, such as darkening of materials, for example. Darkening would lead to charge collection lowering.
- Vacuum: Layer ungluing or outgassing can also impact the charge collection efficiency.
- Radiations and UV: Ionizing and non-ionizing effects can lead to break of polymer chain [20], inducing the darkening of material. Consequently, the material would become less transparent for incident light, which would reduce the charge collection efficiency.

Consequently, the main interesting electro-optical parameter useable to investigate the degradation in optical stack is the quantum efficiency, which reflects the charge collection efficiency.

## 3. Impact of Space Environment on CIS Technologies

### 3.1. Foundry A: Humidity, Thermal Cycling, HTS, UV, Vacuum, and Radiation

Three types of detectors were exposed to several environmental tests. They have the same pixel architecture (same pixel pitch, technological node, and are all front-side illuminated). One is a detector without any optical stack, one has microlenses, and the last is equipped with microlenses and a color filter array (Bayer filter). Microlenses are multilayer polymer PMMA. A SEM view is shown in Figure 1.

Detectors were immersed for 10 min in acetone and 10 min in ethanol. No physical evolution nor delamination was observed.

#### 3.1.1. Humidity and Thermal Cycling

Two detectors of each type were exposed to 500 h at 70 °C and 70% RH + 50 cycles (−55 °C/125 °C) under nitrogen and atmospheric pressure.

**Figure 8:**
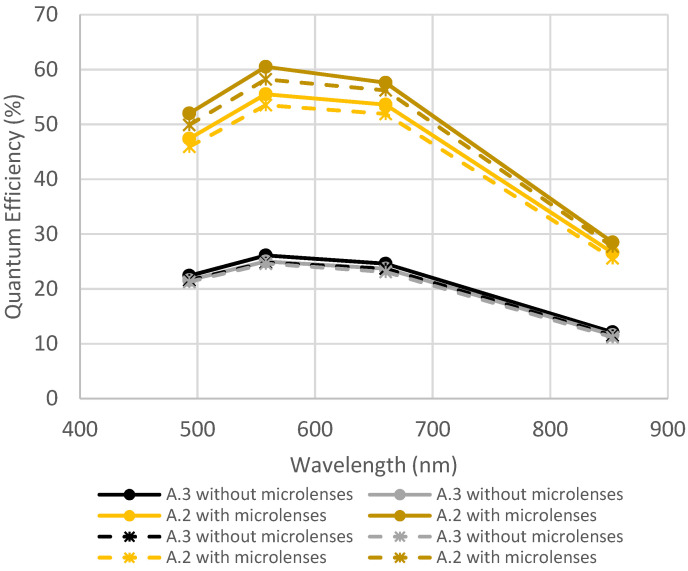
Quantum efficiency before and after thermal cycling + humidity for devices A.2 and A.3. Full lines are initial measurements, and dotted lines are after environmental tests.

**Figure 9:**
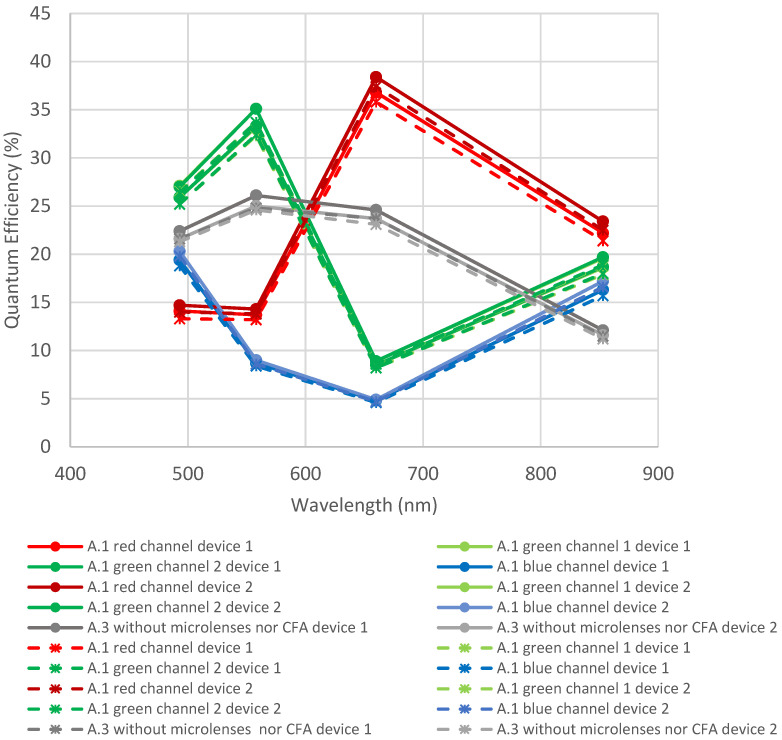
Quantum efficiency before and after thermal cycling + humidity for two devices A.1 and two devices A.3. Full lines are initial measurements, and dotted lines are after environmental tests.

Figure 8 and Figure 9 represent the evolution of quantum efficiency before and after the exposition to thermal cycling and humidity for samples from foundry A. Figure 8 shows a comparison of the design with microlenses (A.2) and without microlenses (A.3). Figure 9 shows a comparison of the designs without microlenses (A.3) and the design with microlenses and CFA (A.1).

Figure 8 shows a slight decrease in QE (<5%), but this remains within the measurement uncertainties and batch-to-batch variations. Indeed, the difference in QE between both samples with microlenses is higher than the degradation due to thermal cycling and humidity.

In Figure 9, the same behavior is noticed: a slight decrease remaining within measurement uncertainties and batch-to-batch variations. Consequently, it can be concluded that CFA is not impacted by thermal cycling and humidity.

#### 3.1.2. High-Temperature Storage

Two detectors with and without microlenses were exposed to 100 °C for 1000 h.

**Figure 10:**
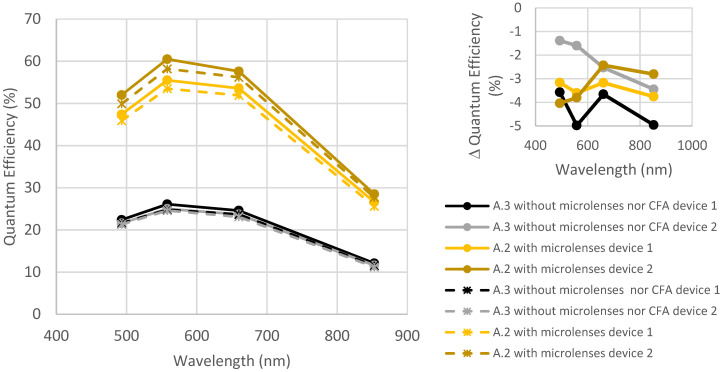
Quantum efficiency before and after HTS. Full lines are initial measurements, and dotted lines are after environmental tests. On the right, the degradation in *QE* is plotted as per Equation (2). Curves are in the same color as devices’ color in the main figure.

Figure 10 represents the degradation in quantum efficiency due to HTS:

(2) $$∆QE\left(\%\right)=\frac{QE_{init}-QE_{final}}{QE_{init}}$$

where *QE_init_* is the quantum efficiency before HTS, and *QE_final_* is the quantum efficiency after exposition to HTS.

As for the previous section, results show a slight decrease in *QE* (<5%), still remaining within measurement uncertainty and lower than lot-to-lot variation. 

#### 3.1.3. UV Insolation 

One detector of each type, i.e., three samples, was positioned in front of a solar simulator ORIEL 300 W for 96 h and was exposed at 0.6 solar unity within the range of 250–450 nm. The optical power contribution was 170.85 W/m^2^.

**Figure 11:**
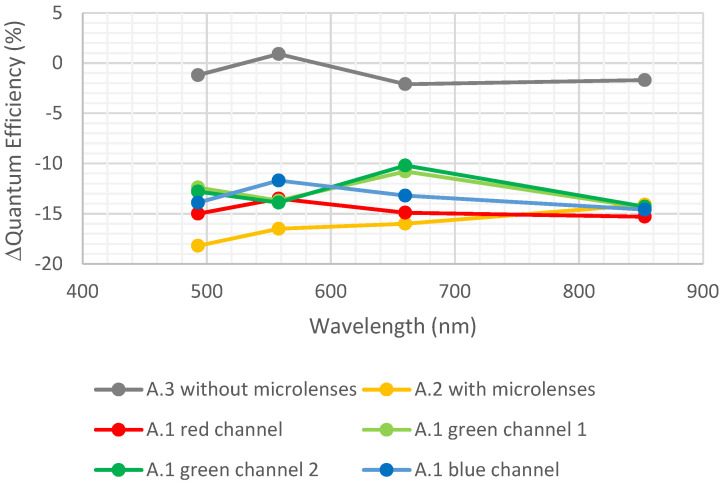
Variation in quantum efficiency due to UV insolation.

Figure 11 represents the degradation in quantum efficiency due to UV insolation calculated as in the previous section.

A degradation in QE (~15%) was observed for the detector containing microlenses. UV insolation may have caused ionization of the PMMA surface, leading to transmission reduction of the microlenses [19]. This could explain this decrease. The same degradation was observed for the detector containing CFA. In the frame of this study, it cannot be concluded that CFAs are immune to UV insolation because photons may not have reached CFA (surface ionization at the microlenses level). Consequently, the robustness of a design with CFA only (no microlenses) cannot be extracted from this work because it is not known if the CFAs were protected by microlenses or if they were really immune to UV insolation. In any case, particular attention has to be paid when designing an optical instrument, and the optical transmission in the UV range has to be carefully studied to protect the optical stack. It is important to notice that sensors dedicated to UV present a dedicated illuminated interface to properly collect the photo-generated charges created very close to the surface, as the absorption coefficient is very high in silicon for such wavelengths. To conclude, the use of color filters and microlenses are not recommended for such UV applications.

#### 3.1.4. Vacuum

One detector of each type was exposed to a vacuum (<10^−6^ Torr) for 1 week.

**Figure 12:**
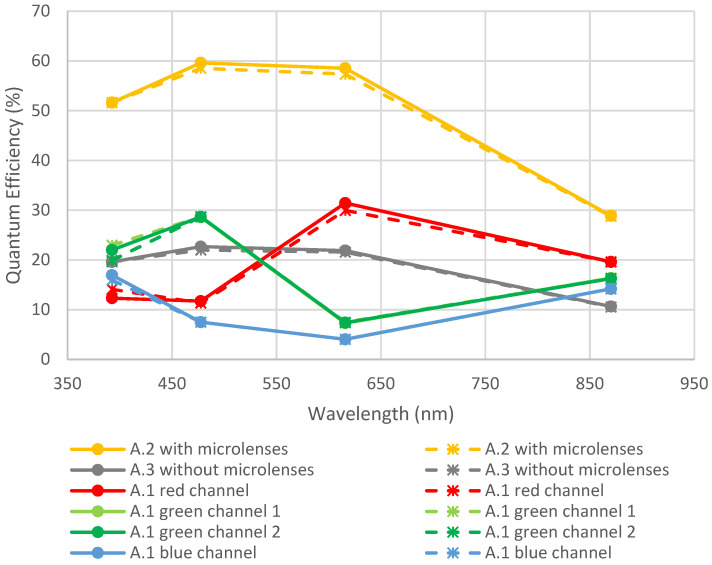
Quantum efficiency before and after vacuum. Full lines are initial measurements, and dotted lines are after environmental tests. Light and dark green curves are superimposed.

Figure 12 represents the quantum efficiency before and after the environmental test.

No degradation was observed, and there were no delamination or outgassing issues with the materials.

#### 3.1.5. Radiations

One sample without optical stack (A.3), one sample with microlenses (A.2), and one sample with microlenses and CFA (A.1) were exposed to 50 MeV protons up to 2 × 10^11^ p/cm^2^.

**Figure 13:**
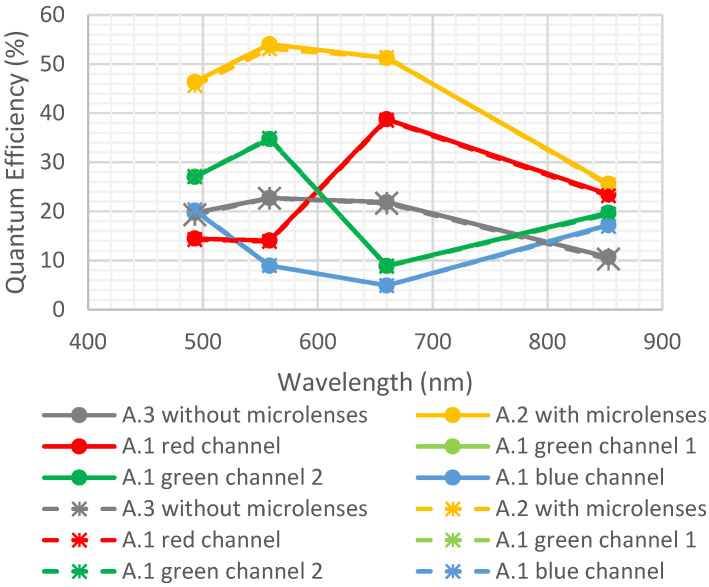
Quantum efficiency before and after proton irradiation. Full lines are initial measurements, and dotted lines are after environmental tests. Light and dark green curves are superimposed.

Figure 13 represents the quantum efficiency before and after irradiation for these devices.

It can be observed that no variation in QE occurred. This means that optical stacks used for these tests are tolerant to non-ionizing doses up to 2 × 10^11^ p/cm^2^.

### 3.2. Foundry B: Humidity, Thermal Cycling, HTS, UV, and Radiation

Several detectors from this foundry were designed in the frame of several projects. They were all front-side illuminated. B.1 and B.2 share the same design, one containing microlenses and one without microlenses. Moreover, design B.4 and design B.5 have pixels with and without color filters on the same matrix [21,22]. Consequently, for these cases, transmittance can be extracted in order to obtain only the degradation in color filter arrays.

#### 3.2.1. Humidity and Thermal Cycling

One detector, B.1, and one detector, B.2, were exposed to 500 h at 70 °C and 70% RH + 50 cycles (−55 °C/125 °C) under nitrogen and atmospheric pressure.

**Figure 14:**
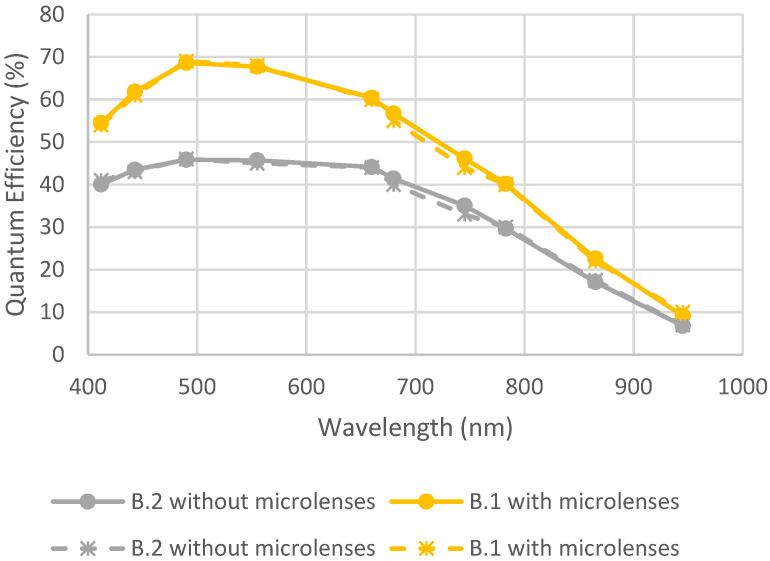
Quantum efficiency before and after humidity and thermal cycling. Full lines are initial measurements, and dotted lines are after environmental tests.

Figure 14 represents the QE for detectors B.1 and B.2 before and after the environmental test. It can be observed that there was no degradation due to humidity and thermal cycling.

#### 3.2.2. High-Temperature Storage

One sample with microlenses (B.1) and one sample without microlenses (B.2) were exposed to 100 °C for 1000 h.

**Figure 15:**
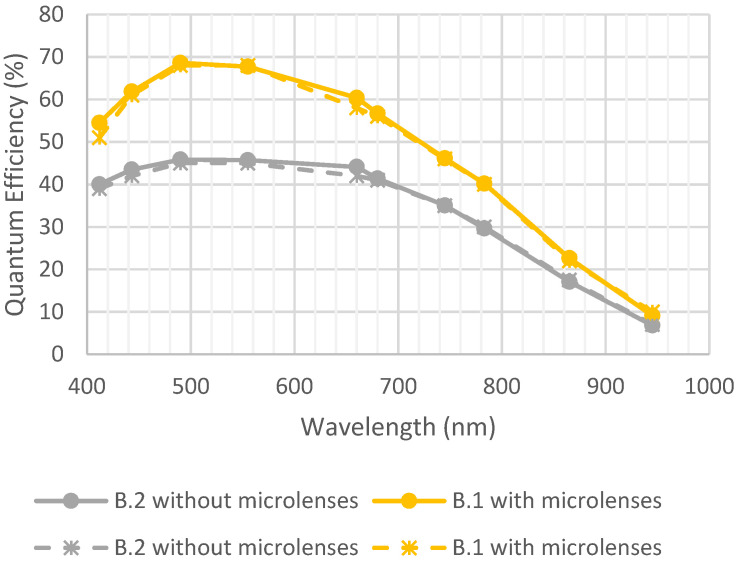
Quantum efficiency before and after high-temperature storage. Full lines are initial measurements, and dotted lines are after environmental tests.

Figure 15 shows the QE for detectors B.1 and B.2. before and after the environmental test. It can be observed that there was no degradation due to high-temperature storage.

#### 3.2.3. UV Insolation 

One sample, B.1, and one sample, B.2, were positioned in front of a solar simulator ORIEL 300 W for 96 h and were exposed at 0,6 solar unity within the range of 250–450 nm. The power contribution was 170.85 W/m^2^.

**Figure 16:**
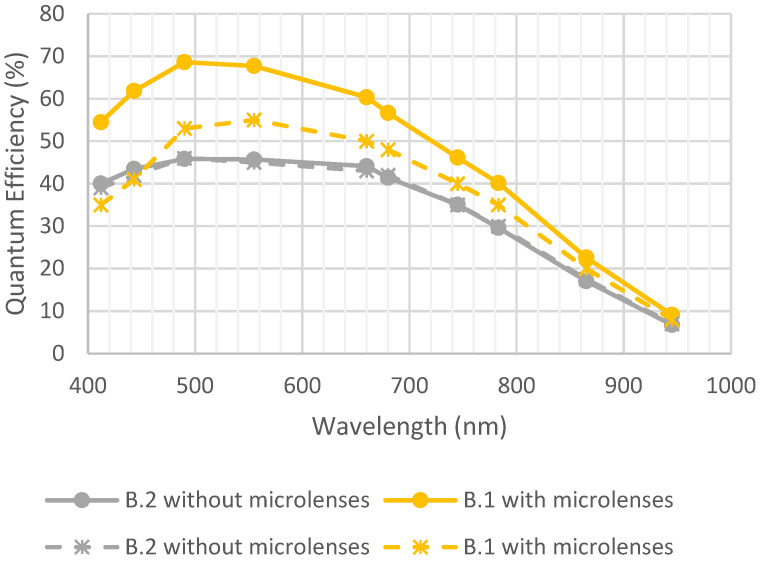
Quantum efficiency before and after UV insolation. Full lines are initial measurements, and dotted lines are after environmental tests.

Figure 16 represents the evolution of QE before and after exposition to UV. For the sample with microlenses, a degradation of around 15% was observed. This result is similar to the degradation observed on devices from foundry A. Moreover, it can be noticed that the quantum efficiency of device B.1 with microlenses after UV insolation was still higher than the quantum efficiency of device B.2 without microlenses before UV insolation. 

#### 3.2.4. Radiations

As per [23], detectors B.3 and B.4 were exposed to gamma radiations up to 100 krad. The irradiation took place at UCL, at room temperature, and the devices were biased. Dose rate was 2 krad/h.

**Figure 17:**
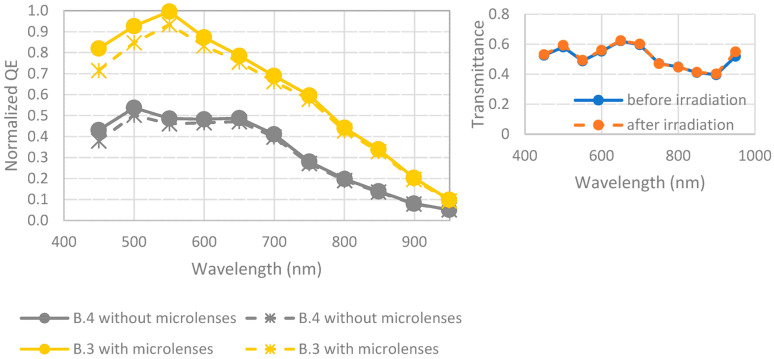
Evolution of quantum efficiency of detectors B.3 and B.4 before and after exposition to gamma radiations (100 krad). Data are from [15]. Full lines are initial measurements, and dotted lines are after environmental tests. On the right, the ratio between devices with and without CFA is shown, demonstrating that the observed degradation is due to the pixel, not the CFA.

Figure 17 represents the evolution of QE before and after irradiation for pixels with or without microlenses. There was the same decrease in QE due to irradiation for samples with and without microlenses, which implies that this degradation comes from the detector, not from microlenses. 

As per [22], detector B.5 was exposed to 600 Mrad using Co60. The irradiation took place at SCK-CEN Brigitte facility, the dose rate was 900 krad/h, and the temperature was 30 °C.

The detector contains areas with CFA and areas without CFA. This helps to determine the contribution of CFA only through transmittance. Consequently, data are not polluted by degradation due to the pixel.

**Figure 18:**
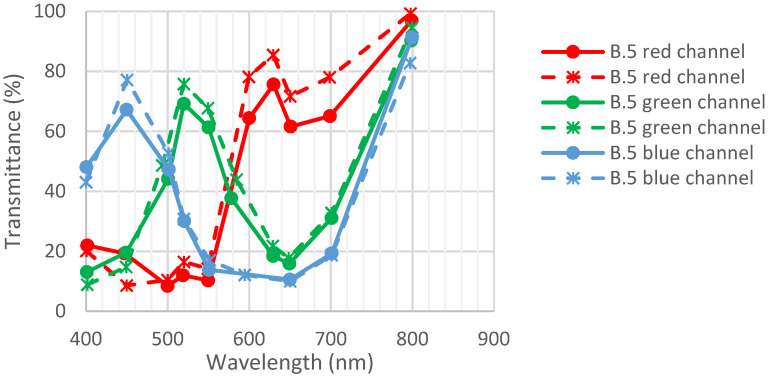
Transmittance of detector B.5 before and after exposition to radiation (600 Mrad). Full lines are initial measurements, and dotted lines are after environmental tests. Data are from [22].

Figure 18 represents the transmittance of CFA before and after irradiation.

**Figure 19:**
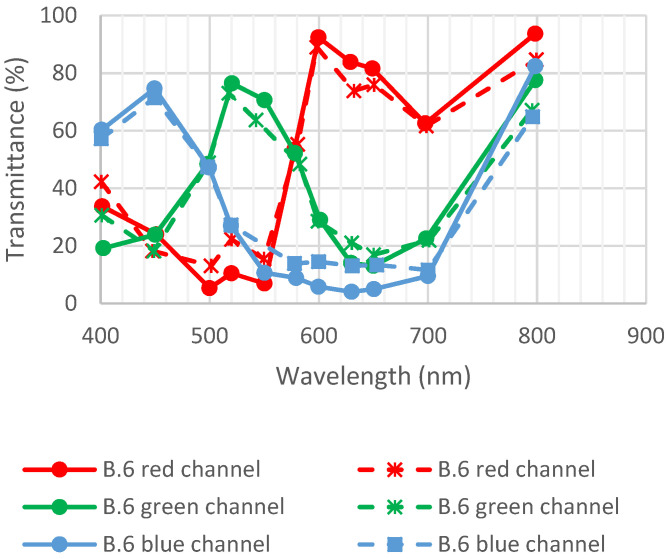
Transmittance of detector B.6 before and after exposition to radiation (1 Grad). Full lines are initial measurements, and dotted lines are after environmental tests. Data are from [21].

As per [21], detector B.6 was exposed to 1 Grad using 10 keV X-rays at CEA, DAM, DIF, and Arpajon. The dose rate was 18 Mrad/h. Figure 19 shows the transmittance of detector B.6 before and after irradiation. No major variation in transmittance was observed, which shows that CFAs are not impacted by irradiation.

### 3.3. Foundry C: Radiations, HTS, Thermal Cycling, and Humidity

Three detectors from foundry C were tested. They all contained microlenses. C.1 and C.2 had CFA, whereas C.3 was the only device containing polarizing filters.

**Figure 20:**
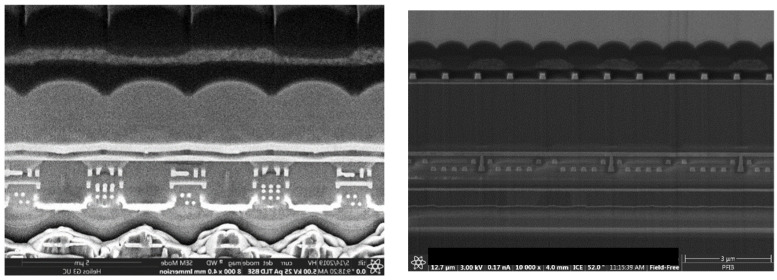
Side view of C.1 (**left**) and C.2 (**right**).

Side views of C.1 and C.2 are given in Figure 20.

**Figure 21:**
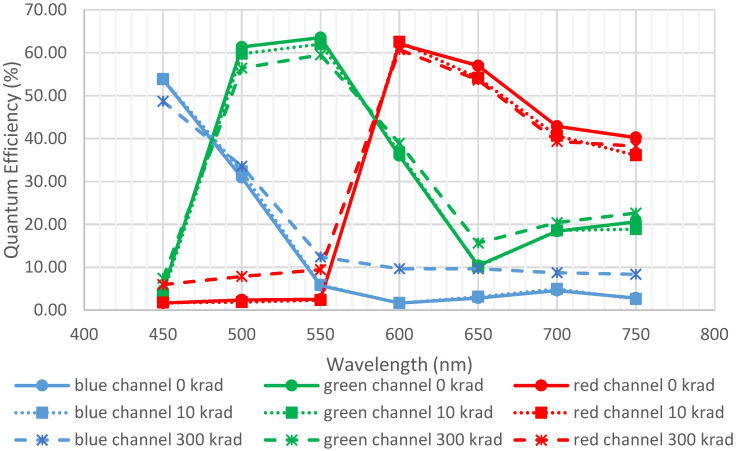
Quantum efficiency for C.1 before and after exposition of protons for two steps (100 krad and 300 krad equivalent doses). Full lines are initial measurements, and dotted lines are after environmental tests.

C.1 was exposed to 49.7 MeV protons at UCL up to 2 × 10^12^ p/cm^2^, leading to a displacement damage dose of 7560 TeV/g. The estimated ionizing dose received was thus 300 krad. A step was also measured at 10 krad. Figure 21 represents the evolution of quantum efficiency before and after irradiation. Data before irradiation come from a datasheet. 

No major degradation was observed. The differences at low QE may come from the fact that measurements were performed at half dynamic. Thus, a low signal was collected by the red channel at a low wavelength and by the blue channel at a high wavelength, inducing a high imprecision in QE measurements for these points. 

Two detectors of C.1 were also exposed to 2000 h at 125 °C (high-temperature storage), two others to 500 cycles at −55 °C/125 °C (thermal cycling), and one sample for 500 h at 70% RH and 70 °C (humidity). The evolution of QE was not observed, but the PhotoResponse Non-Uniformity (PRNU) was the same before and after environmental tests for the same measurement conditions (integration time and flux). Consequently, it can be deduced that there was no major change in the QE.

**Figure 22:**
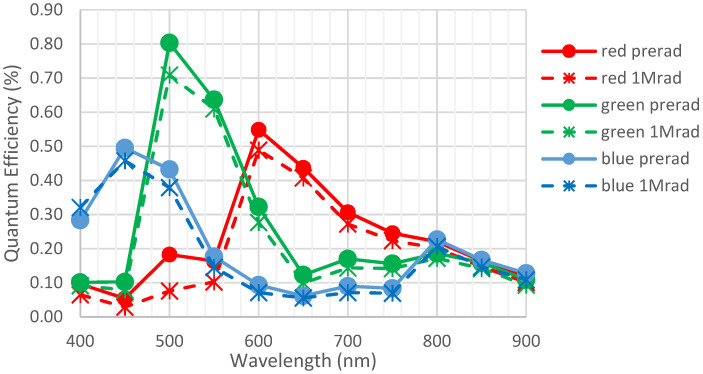
Quantum efficiency of detector C.2 before and after exposition to 1 Mrad. Full lines are initial measurements, and dotted lines are after environmental tests.

C.2 was exposed to gamma rays up to 1 Mrad at TRAD, Toulouse, France. Figure 22 shows the evolution of the quantum efficiency before and after irradiation. It can be observed that there was a slight drop in quantum efficiency. At this dose level, the measurement becomes more difficult because of photodiode degradation, but the performance due to the optical stack is still higher than the performance of an imager without an optical stack.

C.3 was the only detector containing polarizing filters. Some literature exists about this optical stack, but they have not been exposed to high constraints [3,24,25]. 

Three detectors of C.3 were exposed to gamma rays up to 50 krad at TRAD, Toulouse, France. The dose rate was 310 rad/h.

**Figure 23:**
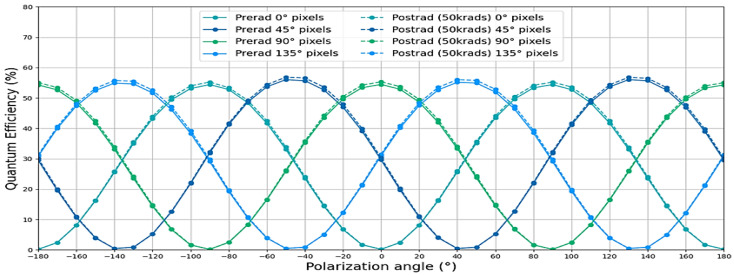
Evolution of quantum efficiency of C.3 at 660 nm in function of polarization angle before and after irradiation.

Figure 23 shows the evolution of The QE according to the polarization angle before and after irradiation. The wavelength for this measurement is 660 nm. The same results were obtained at 470 nm and 850 nm. The QE of device C.4 was at its highest when the light polarization angle was equal to the pixel’s filter polarization angle. When the two angles were orthogonal, the QE dropped to zero. No evolution of QE according to irradiation was observed.

### 3.4. Foundry D: Thermal Cycling, Humidity, and HTS

Two detectors from foundry D were also exposed for 2000 h at 125 °C (high-temperature storage), two others to 500 cycles at −55 °C/125 °C (thermal cycling), and one sample for 500 h at 70% RH and 70 °C (humidity). 

The evolution of QE was not observed, but the PhotoResponse Non-Uniformity (PRNU) was the same before and after environmental tests for the same measurement conditions (integration time and flux). Consequently, it can be deduced that there was no major change in the QE.

### 3.5. Foundry E: Radiations

One sample from foundry E was exposed to gamma irradiation up to 100 Mrad, with a dose rate of 300 krad/h at room temperature [26].

**Figure 24:**
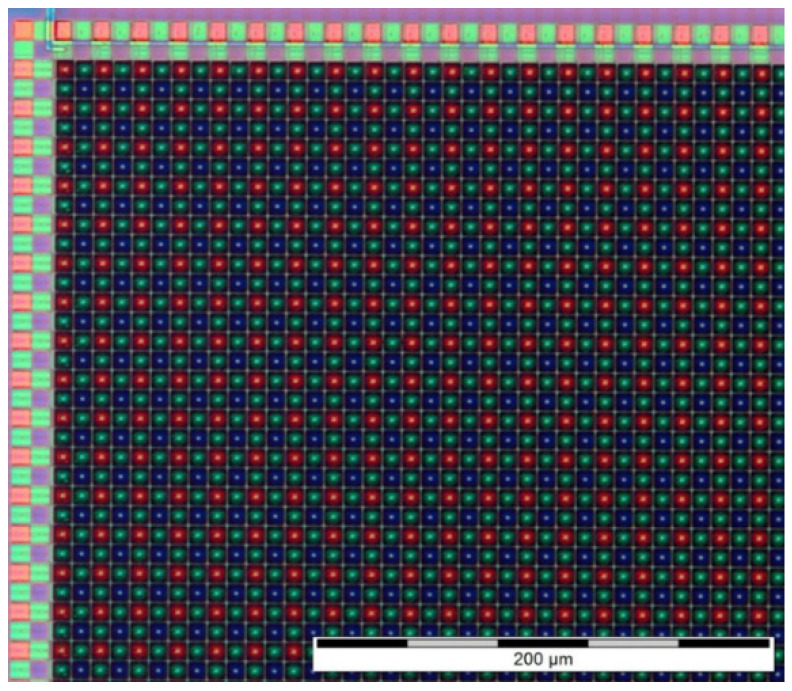
Top view of detector from foundry E.

Figure 24 represents a top view of the detector.

**Figure 25:**
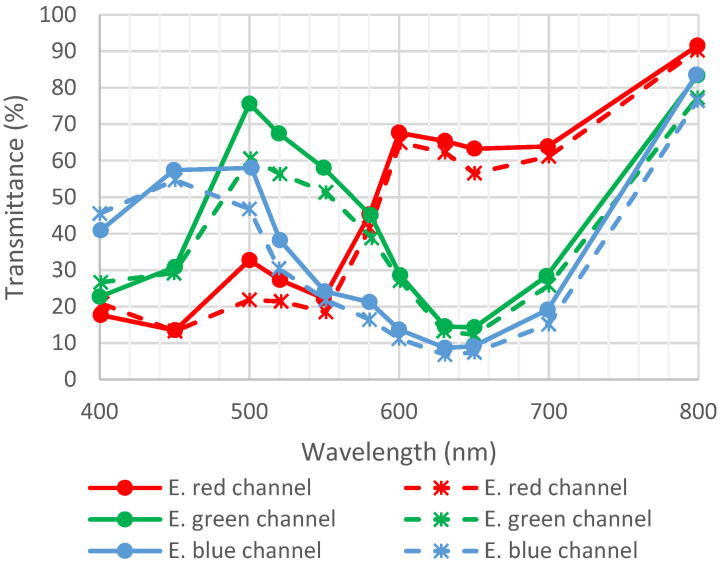
Evolution of quantum efficiency of detector E before and after exposition to gamma radiations (100 Mrad). Full lines are initial measurements, and dotted lines are after environmental tests.

The result of the radiation campaign is given in Figure 25. Some degradation was observed, but at 100 Mrad, the uncertainty due to measurement was high. Consequently, the variation does not seem critical compared to the gain in performance due to microlenses.

### 3.6. Foundry F: Radiation

Detector from foundry F was exposed to 49.7 MeV protons up to 1 × 10^11^ p/cm^2^ and to Co60 up to 10 krad.

As for other detectors, no critical variation was observed before and after irradiation. 

## 4. Discussion

Many devices have been exposed to a wide range of harsh environments. Results are summarized in Table 3. It can also be noticed that during all the campaigns, no visual defects were observed.

These constraints have permitted us to anticipate potential degradation during the lifetime:

- Thermal cycling/humidity: no visual defects have been observed, and there was no significant degradation in quantum efficiency (results remain within the measurement uncertainties). This means that optical stacks have not been impacted, no ungluing nor weakening occurred due to thermal cycling, and no mechanical distortion nor modification of layers occurred due to humidity.
- HTS: No noticeable variation in QE was observed, which means that optical modification occurred. Optical stacks are suitable for long-term applications.
- Vacuum: No variation in behavior was observed due to vacuum exposition. Thus, no ungluing nor outgassing occurred.
- Radiations: Small variations were observed for devices C.2 and E, but the total cumulative dose of radiation tests were higher than the usual space mission’s needs (more than 1 Mrad). For other devices, no clear radiation damage was observed up to the maximum total dose.
- UV: Some ionizing effects were observed, leading to a decrease of 15% in quantum efficiency. This may be due to the darkening of the material. However, this decrease (~15%) is lower than the gain due to the addition of an optical stack (+50% at minimum from Figure 7). In other words, the QE of designs with microlenses after the environmental test is still higher than the QE of designs without microlenses before the environmental test. Optical instrument designers have to be careful with the UV range when designing the system.

No major changes were observed in terms of the QE for most of the environmental tests. The main impact occurs with UV insolation, where a decrease of 15% was noticed for designs containing microlenses. This may be due to a darkening of microlenses, which prevents a good charge collection. Tests were conducted at ambient pressure, which is not necessarily a worst-case scenario for space applications. However, it can be pointed out that this observed degradation is lower than the gain due to the addition of an optical stack, which can bring more than a 50% gain in QE (see Figure 7 for example).

Zanella et al. [9] obtained similar results: tested microlenses successfully passed environmental tests but exhibited limited UV stability, where the material lost transmission.

Other work [27] has seen a degradation in spectral response due to radiations after 1 Mrad for a detector containing CFA. It seems that there was no design without CFA; thus, it is not known if all the degradation is due to CFA only or also to photosentive element or other optical elements in the optical stack. The CFA used for this study was made with resin and organic pigments, which is not the case in the frame of this study. 

Consequently, a large set of devices coming from several foundries have been exposed to a large range of environmental tests. Optical stacks can handle such constraints, meaning that the detection chain will probably be more limited by the detector itself than the optical stack.

## 5. Conclusions

Several devices from several foundries and several optical stacks were exposed to heavy constraints, which are a worst-case scenario compared to most space environments.

This work has highlighted that optical stacks such as microlenses, CFA, or polarizing filters from commercial CMOS foundries can be compatible with space applications. The gain in performance that an optical stack can bring is higher than the degradation induced by environmental constraints (radiations, temperature storage, thermal cycling, UV, and vacuum). Thus, the dimensioning element in the detection chain for space application would probably be the detector itself, more than the optical stack. Particular attention has to be paid to the design of optical instruments in the UV range.

## Figures and Tables

**Table 1:** Devices analyzed (Y = Yes, N = No, FSI = Front-Side illuminated, BSI = Back-Side Illuminated, FEOL = Front End of Line, BEOL = Back End of Line). Background color used to delimit devices from a same foundry. The letter represents the foundry (A to F), and the number represents the number of the device.

Foundry.Device	Technology Node (nm)	Pixel Pitch (µm)	Illumination	Microlenses	Color Filter Array	Polarizing Filters
A.1	180	5.5	FSI	Y	Y	N
A.2	180	5.5	FSI	Y	N	N
A.3	180	5.5	FSI	N	N	N
B.1	180	5	FSI	Y	N	N
B.2	180	5	FSI	N	N	N
B.3	180	7	FSI	Y	N	N
B.4	180	10	FSI	N	N	N
B.5	180	10	FSI	N	Y	N
B.6	180	10	FSI	N	Y	N
C.1	65	3.45	FSI	Y	Y	N
C.2	65	1.1	BSI	Y	Y	N
C.3	65	3.45	FSI	Y	N	Y
D	110	2.8	FSI	Y	Y	N
E	180	8.5	FSI	Y	Y	N
F	90 FEOL	13/26/52	FSI	Y	N	N
	65 BEOL	13/26/52				

**Table 2:** List of environmental tests performed on detectors.

	UV	Thermal Cycling/Humidity (RH: Room Humidity)	High-Temperature Storage (HTS)	Vacuum	Radiations
Foundry A	96 h at 0.6 solar unity	500 h at 70 °C and 70% RH + 50 cycles (−55 °C/125 °C) under nitrogen and atmospheric pressure	1000 h at 100 °C	1 week at <10^−6^ Torr	50 MeV protons up to 2 × 10^11^ p/cm^2^
Foundry B	96 h at 0.6 solar unity (B.1 and B.2)	500 h at 70 °C and 70% RH + 50 cycles (−55 °C/125 °C) under nitrogen and atmospheric pressure (B.1 and B.2)	1000 h at 100 °C (B.1 and B.2)		Co60 100 krad (B.3 and B.4) Co60 600 Mrad (B.5) 10 keV Xrays 1 Grad (B.5)
Foundry C		500 cycles −55 °C/125 °C + 500 h at 70% RH and 70 °C (C.1)	2000 h at 125 °C (C.1)		49.7 MeV protons up to 2 × 10^12^ p/cm^2^ (C.1) Co60 1 Mrad (C.2) Co60 50 krad (C.3)
Foundry D		500 cycles −55 °C/125 °C + 500 h at 70% RH and 70 °C	2000 h at 125 °C		
Foundry E					Co60 100 Mrad
Foundry F					49.7 MeV protons up to 1 × 10^11^ p/cm^2^ Co60 10 krad

**Table 3:** Summary of results obtained.

	UV	Thermal Cycling/Humidity	HTS	Vacuum	Radiation
Foundry A	~15%	<5%	<5%	<5%	<5%
Foundry B	~15%	<5%	<5%		<5%
Foundry C		No major change (C.1)	No major change (C.1)		~5% (C.1) ~10% (C.2) <5% (C.3)
Foundry D		No major change	No major change		
Foundry E					<10%
Foundry F					<5%

## Data Availability

No raw data available.
